# Fabrication and Modeling of Matching System for Air-Coupled Transducer

**DOI:** 10.3390/mi13050781

**Published:** 2022-05-17

**Authors:** Jinjie Zhou, Jiaqi Bai, Yao Liu

**Affiliations:** School of Mechanical Engineering, North University of China, Taiyuan 030051, China; zhoujinjiechina@126.com (J.Z.); baihuahua6@outlook.com (J.B.)

**Keywords:** air-coupled acoustic transducer, matching layer, acoustic impedance, bonding layer

## Abstract

The tremendous acoustic impedance difference between the piezoelectric composite and air prevents the ultrasonic transition, resulting in low amplitude for the received signal for the composite defect detection using an air-coupled transducer. The matching system, which includes the matching layers and bonding layers attached to the piezoelectric composite, can reduce the acoustic impedance difference and benefit the acoustic transition. In this paper, the fabrication method and modeling for the matching layers are proposed to optimize the transducer performance. The effects of bonding layer material on the transducer performance are also discussed. Experiments were conducted for modeling validation. The proposed model can predict the matching layer acoustic properties with an error of less than 11%. The bonding layer using the same material as the first matching layer can help to increase the sensitivity by about 33% compared to the traditional epoxy bonding. The optimized air-coupled ultrasonic transducer, based on the results of this study, has a 1283 mV amplitude in the air, which is 56% higher than commercially available transducers, and can identify the defects in two typical non-metallic composite materials easily.

## 1. Introduction

The air-coupled acoustic transducer uses air as the medium to detect the defect in aerospace composites, foods, drugs, etc. [1,2], in which the coupling agent is prohibited or caution used. An air-coupled acoustic transducer commonly uses the piezoelectric composite as the acoustic wave source. When the acoustic wave propagates from the piezoelectric composite to the air directly, a nearly total reflection occurs on the interface due to the tremendous acoustic impedance discrepancy between the piezoelectric composite and air. The high reflection ratio of the acoustic wave limits the energy into the air and tested material, resulting in a low signal amplitude [3,4]. In order to solve this problem, the transition/matching layers are often attached to the piezoelectric composite for the acoustic transmission [5]. The properties of the matching layer, including the geometric and acoustic parameters, ultimately determine the acoustic transmission process [6].

The study of matching layers for the air-coupled transducer has been reported. Toda et al. [7] conducted impedance matching by adjusting the air space and reflectivity by insertion a reflective layer between the transducer and the propagation medium. Kelly et al. [8] added a porous material with extremely low impedance and the low-density rubber material as the matching layer, which induced the amplitude of the received signal to increase by 30 dB compared with the unmatched one. Tomas et al. [9,10] proposed a better matching configuration by studying the acoustic properties of polyethersulfone and other materials to obtain better sensitivity and bandwidth transducer. Saito et al. [11] optimized the acoustic impedance of the matching layer by using the transmission line model, which was proved by experiments to increase the sensitivity by 20 dB by using silicon rubber and thermoplastic hollow microspheres mixture as a matching layer. Botun et al. [12] developed an air-coupled ultrasonic transducer with a simple structure and high sensitivity by using honeycomb polypropylene iron electret film as a matching layer. Kazys et al. [13,14] used low impedance polystyrene foam to improve the efficiency, bandwidth, and radiation pulse waveform of the PMN-32PT crystal transducer. Guo et al. [15] analyzed the effect of the matching layer material on the vibration mode shape of the transducer and found the resin epoxy can improve the transmission ratio. Wang et al. [16] used the superposition of two low impedance matching layers to improve the sensitivity of the designed transducer, which can detect microcracks. Wu et al. [17] developed a single matched layer air-coupled ultrasonic transducer using a hollow polymer microspheres/epoxy resin system, increasing the transducer sensitivity by 20.9 dB. Song [18] introduced the periodic subwavelength apertures that employ coupled resonances to enhance the efficiency and bandwidth of the non-contact ultrasonic transducers. Based on the literature review above, the matching layer properties, including the material and acoustic properties, have a significant influence on the sensitivity of the air-coupled transducer. However, the modeling of the matching layer properties and the bonding material effects on the matching layers is currently missing.

In this study, a double-layer matching system on the air-coupled transducer was investigated. The matching theory of the acoustic impedance is firstly introduced. Then modeling to predict the density, acoustic transmission speed, and the acoustic impedance of the matching layer is proposed based on the raw material. Experiments to validate the modeling results and reveal bonding material effects on the transducer sensitivity are introduced. Lastly, the optimized transducer was applied to detect the defects in two typical non-metallic materials to prove the feasibility.

## 2. Matching Theory and Modeling of Acoustic Impedance

### 2.1. Matching Theory of Acoustic Impedance 

A material acoustic impedance *Z* can be calculated by the density ρ and acoustic propagation speed *c_L_* in the material, as shown in Equation (1) [19].

(1) $$Z=\rho c_{L}$$

Assuming the piezoelectric composite, medium, and tested sample are half infinite. The acoustic impedance of piezoelectric composite, the Nth matching layer, and medium are represented by *Z*_0_, *Z_n_*, and *Z_L_*, respectively. The theoretical acoustic impedance for single matching layer Z_1_ can be calculated by Equation (2) [19]:

(2) $$Z_{1}=\sqrt{Z_{0}Z_{L}}$$

The theoretical acoustic impedances for double-layer matching can be calculated as Equations (3) and (4) [19] for the first and second matching layers.

(3) $$Z_{1}=\sqrt[{4}]{Z_{0}^{3}Z_{L}}$$

(4) $$Z_{2}=\sqrt[{4}]{Z_{0}Z_{L}^{3}}$$

where *Z*_1_ and *Z*_2_ are the acoustic impedances for the first and second matching layers.

The ultrasonic wave generated by the piezoelectric composite propagates as a simple harmonic wave to the matching layer. In order to ensure a continuous ultrasonic oscillation, the matching layer thickness should be a quarter of the wavelength, which can also reduce the ultrasonic attenuation. Based on the theory above, the matching layer thickness should be:

(5) $$b=\frac{c}{4f}$$

where *c* is the acoustic propagation speed, *f* is the frequency of the acoustic, and *b* is the thickness of the matching layer.

### 2.2. Modeling of Matching Layer Acoustic Impedance

Assuming the matching layer consists of the N components, with the weight of *m*_1_, *m*_2_, *m*_3_, …, and *m*_N_. The corresponding densities for the N components are *ρ*_1_, *ρ*_2_, *ρ*_3_, …, and *ρ*_N_, respectively. Generally, the matching layer uses the curing process to form the solid shape from liquid, which may cause the volume change. The volume variation for the N components, described by the shrinkage or expansion ratio, are *k*_1_, *k*_2_, *k*_3_, …, and *k*_N_, where the negative value means the expansion and the positive value means shrinkage. The volume of the matching layer after solidification is

(6) $$V=\sum_{i=1}^{N}\left(1-k_{i}\right)\frac{m_{i}}{\rho_{i}}$$

The density of the matching layer is

(7) $$\rho =\frac{\sum_{i=1}^{N}m_{i}}{V}=\frac{\sum_{i=1}^{N}m_{i}}{\sum_{i=1}^{N}\left(1-k_{i}\right)\frac{m_{i}}{\rho_{i}}}$$

In order to model the acoustic velocity of the matching layer, the acoustic velocity in each pure component in solid mode should be measured in advance. Assuming the acoustic velocity in N components are *v*_1_, *v*_2_, *v*_3_, …, and *v*_N_. Based on the probability statistics theory, when the matching layer thickness is *b*, the average prorogation distance, in statistics, of acoustic in the *i*^th^ component is

(8) $$b_{i}=b\frac{(1-k_{i})m_{i}/\rho_{i}}{V}$$

The propagation time in the *i*^th^ component is *t*_i_ = *b*_i_/*v*_i,_ and the total time used to cross the matching layer is

(9) $$t=\sum_{i=1}^{N}\frac{b_{i}}{v_{i}}$$

So the average acoustic velocity in the matching layer is

(10) $$v=\frac{b}{t}=\frac{\sum_{i=1}^{N}\left(1-k_{i}\right)\frac{m_{i}}{\rho_{i}}}{\sum_{i=1}^{N}\left(1-k_{i}\right)\frac{m_{i}}{\rho_{i}v_{i}}}$$

Based on the Equation (1), the acoustic impedance of the matching layer is

(11) $$Z=\rho v=\frac{\sum_{i=1}^{N}m_{i}}{\sum_{i=1}^{N}\left(1-k_{i}\right)\frac{m_{i}}{\rho_{i}}}\frac{\sum_{i=1}^{N}\left(1-k_{i}\right)\frac{m_{i}}{\rho_{i}}}{\sum_{i=1}^{N}\left(1-k_{i}\right)\frac{m_{i}}{\rho_{i}v_{i}}}=\frac{\sum_{i=1}^{N}m_{i}}{\sum_{i=1}^{N}\left(1-k_{i}\right)\frac{m_{i}}{\rho_{i}v_{i}}}$$

Based on Equation (11), with the mass, density, shrinkage/expansion ratio, and acoustic velocity of each component, the acoustic impedance of the matching layer can be calculated.

## 3. Fabrication of Transducer

From the literature [8,11,13,17], the first matching layer is commonly fabricated by the mixture of hollow glass microspheres with epoxy, and the second matching layer is often made of microcellular foam polypropylene. Their acoustic impedances can be adjusted by changing the ratio between hollow glass microspheres and epoxy and the foaming rate, respectively. In this study, the matching layer used the same raw materials.

### 3.1. 1-3 Piezoelectric Composite

The piezoelectric composite used in this study is the 1-3 type, which consists of one dimension piezoelectric ceramic column and a 3D polymer structure. The embed polymer helps to reduce the density and acoustic impedances of the piezoelectric composite due to the low density and acoustic impedances. The reduced acoustic impedance of the composite makes it an ideal material for an air-coupled piezoelectric transducer [9,20] to ensure more wave energy can propagate into the air. The 1-3 type piezoelectric ceramic/polymer has the following advantages for the air-coupled transducer:

(1) Low acoustic impedance, which is between the pure piezoelectric ceramic and polymer, and easier to achieve the impedance matching.

(2) High electromechanical coupling factor, which is almost the same as the longitudinal electromechanical coupling coefficient of piezoelectric ceramic when the composite is working under the thickness mode.

(3) The mechanical quality factor is low, which is suitable for the broadband transducer.

(4) Weak lateral coupling effects due to the separation of the polymer, which is suitable for the longitudinal transducer.

For the machining of piezoelectric composite, the solid piezoelectric ceramic need to dice or wire saw some kerfs on the surface to separate the ceramic block into the small individual columns. Then the polymer is immersed and cured on the kerf to connect the individual columns to a whole part. After the curing, the top and bottom of the composite surface are ground to remove the uncut ceramic layer and the excess polymer layer. Then continuing grinding is conducted to obtain the specified thickness of the composite.

### 3.2. Fabrication of the First Matching Layer

**Figure 1:**
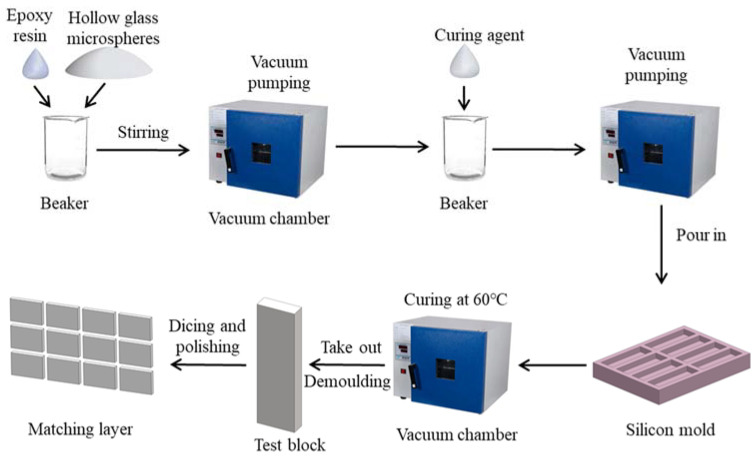
The steps to fabricate the matching layer.

The first matching layer consists of hollow glass microspheres, epoxy, curing agent, and diluent. The density of the hollow glass microsphere changes with the diameter; the smaller the diameter of the hollow glass microsphere, the larger density is. The density and acoustic velocity of the first matching layer can be adjusted by changing the hollow glass microsphere’s diameters and weight ratios. The fabrication method is mold casting in a vacuum chamber, which is shown in Figure 1:

Step 1. The epoxy and curing agents are weighted in 6:1, suggested by the vendor (Shanghai Aotun Chemical Technology Co., LTD, Shanghai, China), by analytical balance and poured into a glass beaker for a mixture. Then the hollow glass microspheres are weighted based on the designed weight ratio and added to the beaker. If needed, the diluent weighted in the ratio of the epoxy and curing agent is added to assist the air bubble release.

Step 2. After all raw materials are put in the beaker, a stirring of about 5 min is taken for the mixture. The vacuum-pumping process under the pressure of −0.1 Mpa is applied for 5 min to help the evacuation of air bubbles.

Step 3. The mixture is then poured into the rectangular mold, followed by another 5 min vacuum to remove the bubbles and moisture.

Step 4. Curing on a thermostat at 60 °C temperature for 12 h.

Step 5. After curing, the composite is taken from the mold and cut into the same size as the 1-3 piezoelectric composite.

Step 6. The density and acoustic velocity of the matching layer are determined by using drainage and pulse insertion

Step 7. Cut the matching layer thickness to a quarter of the acoustic wavelength from the calculation.

### 3.3. Fabrication of Second Matching Layer

The second matching layer needs a low theoretical acoustic impedance and is cost-effectively machined to the designed thickness. Aerogel has low acoustic impedance, but its machinability and bonding properties are poor [21]. In this study, the second matching layer is made from polypropylene foam. It has a low density and a high bubble ratio leading to a low acoustic impedance, and can be considered as a composite of the polypropylene and air microsphere. The foaming rate controls the matching layer acoustic properties.

### 3.4. Transducer Assembly

**Figure 2:**
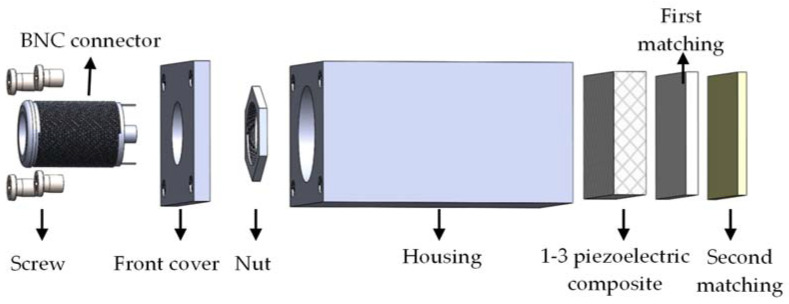
Structure of air-coupled transducer.

Figure 2 shows the transducer design in this study. The front and rear surfaces of 1-3 piezoelectric composite are coated with metal films as the electrodes. On the front surface of 1-3 piezoelectric composite, the first and second matching layers are bonded to form the core of the transducer. A standard BNC connector is screwed to the housing through the front cover. BNC connector links the wire of the 1-3 piezoelectric composite to receive and send the electrical signal from the outside device.

## 4. Experiment Setup

**Figure 3:**
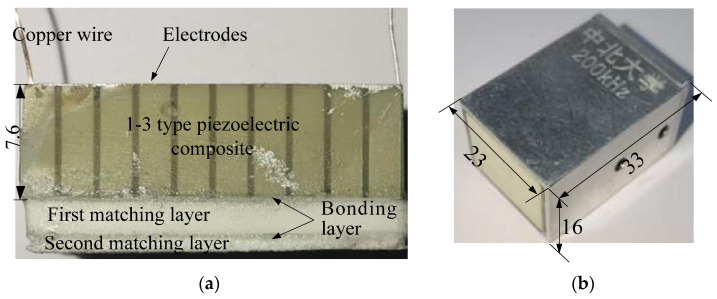
Air-coupled ultrasonic transducer. (**a**) 1-3 piezoelectric composite with double matching layer and (**b**) self-developed transducer.

The PZT-4 ceramics (Shandong Weifang Jude Electronics Co. Ltd., Shanghai, China) was used as the piezoelectric composite due to the high electromechanical coupling factor, which can generate more ultrasonic waves with a provided energy. A 20 × 14 × 8 mm cuboid PZT-4 was cut by the dicing machine (SYJ-400, Shenyang Kejing, Shenyang, China) to create the 0.4 mm width and 7.8 mm depth kerf grid on the surface. The parallel distance of kerfs was 2.4 mm which can create 2 mm × 2 mm PZT-4 columns on the surface after dicing. The epoxy (E51, Shanghai Aotun Chemical Technology Co., LTD, Shanghai, China) was poured on the PZT-4 to fill the kerfs and connect the columns. The composite was put in a vacuum chamber to remove air from the composite. After 24 h curing, the composite was ground by a surface grinder (M7230H, Hangzhou grinding machine Co. Ltd., Hangzhou, China) to remove the uncut layer on the bottom and epoxy on the front surface. The ultimate thickness of the composite controlled by grinding was 7.6 mm to match the 200 kHz resonance frequency. The density and acoustic propagation velocity of the 1-3 type piezoelectric composite were measured by the drainage and pulse insertion method, which were 5501.81 kg/m^3^ and 3521 m/s, respectively. Based on Equation (1), the 1-3 piezoelectric composite acoustic impedance was 19.37 MRayl. Table 1 summarizes the 1-3 piezoelectric composite properties. The front and bottom surfaces 1-3 piezoelectric composite were coated with 50 nm silver via chemical vapor deposition to generate the positive and negative electrodes. The electrode surfaces were welded to two copper wires, as shown in Figure 3a.

From the matching theory mentioned in Section 2, for the double-layer matching system, the acoustic impedance of the first and second matching layers should be 1.32 and 0.0062 MRayl, respectively. In order to achieve the acoustic impedance needed for the first layer, a series of matching layers were fabricated. The first layer consisted of the hollow glass microspheres and E51 epoxy resin. In order to identify the effects of fabrication parameters on the matching layer’s acoustic properties, especially in acoustic impedances, the matching layers with different glass microsphere sizes and weight ratios were made. Table 2 shows the parameters of the hollow glass microspheres used in this study, which have an average diameter of 100, 85, and 70 μm. The glass microsphere weight ratios of 10%, 15%, 20%, and 30% were experimental studied to help the modeling process. In order to facilitate the mixture and curing process, the diluent (butyl glycidyl ether) was added, if necessary, when the bubble could not be expelled completely. After the fabrication process, the matching layer’s density and acoustic velocity were measured to calculate the acoustic impedances. The experiment results were used to validate the proposed matching layer modeling. The exact weight ratios of the first and second matching layers based on the calculated theoretical acoustic impedances were provided. After the determination of the recipe of the matching layer, the first and second matching layers were fabricated, and the acoustic parameters were tested.

The influence of the bonding layer on acoustic propagation and transducer performance is also discussed in this study. In order to connect the 1-3 piezoelectric composite, first matching layer, and second matching layer, the bonding layer should be added to the contact surfaces. Table 1 also lists three types of the bonding layer to be used in the experiment to identify the potential effect.

The first matching layer, bonding layer, and second matching layer, after determination, were attached to the surface of 1-3 piezoelectric composite to form the core of the transducer, as seen in Figure 3a. An aluminum housing enclosed the core, and a BNC connector was on the front surface of the transducer to link the 1-3 piezoelectric composite and outside device. Figure 3b. shows the transducer by using the 1-3 piezoelectric composite, first matching layer, bonding layer, and second matching layer fabrication in this study.

**Figure 4:**
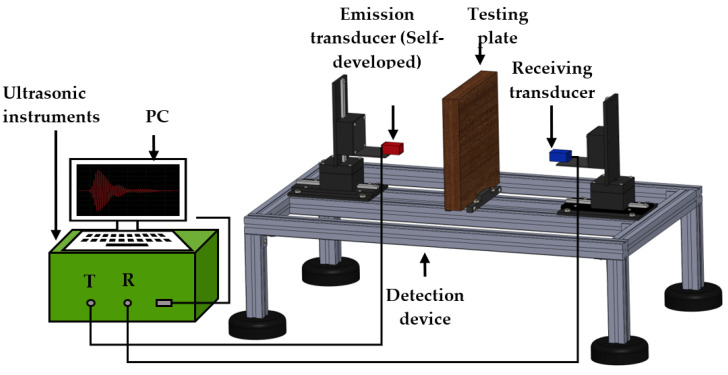
Testing platform.

In order to validate the proposed matching layer modeling and study the effect of bonding layer properties on the transducer performance, a testing platform was built, as presented in Figure 4. The self-developed 200 kHz transducers and a commercially available one (0.2 K 14 × 20 N-TX, Japan Probe Co. Ltd., Japan) were put on the two ends of a linear stage to emit and receive the acoustic signal. Air and testing plate were in the middle of the transducers to act as the medium and inspected material. Both transducers are connected with ultrasonic instruments and computers to control the testing process. The exciting signals output from the self-developed ultrasonic instruments were 200 kHz Hanning windowed three-cycle sine bursts with V_pp_ of 138 V. The received signals were filtered and enveloped to obtain the amplitude. The comparisons of amplitude were conducted to evaluate the performance of the transducer. In this study, the plate was not used first to validate the influence of different matching layers and bonding layers on the transducer sensitive for the modeling and theory validation. Two types of plates (CFRP and PVC foam) with and without artificial defects were put in the middle of the transducers to emphasize the practical application of the transducer. The sizes for the CFRP and PVC foam were 540 mm × 500 mm × 5 mm and 200 mm × 200 mm × 30 mm, respectively. The artificial defects were created by milling at the bottom of the tested materials with the diameter and depth of Φ 5 × 3 and Φ10 × 8 mm for the CFRP and PVC foam, respectively.

## 5. Results and Discussions

### 5.1. Effect of Material Properties on Acoustic Impedance

Table 3 lists the measured acoustic properties of the first matching layers with different glass microsphere diameters and weight ratios. The data of the BR20 glass microsphere in the 30% weight ratio are not reported due to the high viscosity, which prevented the air bubble extraction even with the diluent and failed the homogeneous acoustic properties of the matching layer. Based on the results in Table 3, the higher the hollow glass microsphere weight ratio is, the lower the density and longitudinal wave velocity are. From the microstructure of the first matching layer, the higher hollow glass microsphere weight ratio means that more space is filled by the air inside of the microsphere and longer time the acoustic wave takes to propagate in the glass microsphere, which decreases the overall density and acoustic velocity. With the decrease in the hollow glass microsphere diameter under the same weight ratio, both the density and velocity increase due to the increase in the glass weight percentage.

**Figure 5:**
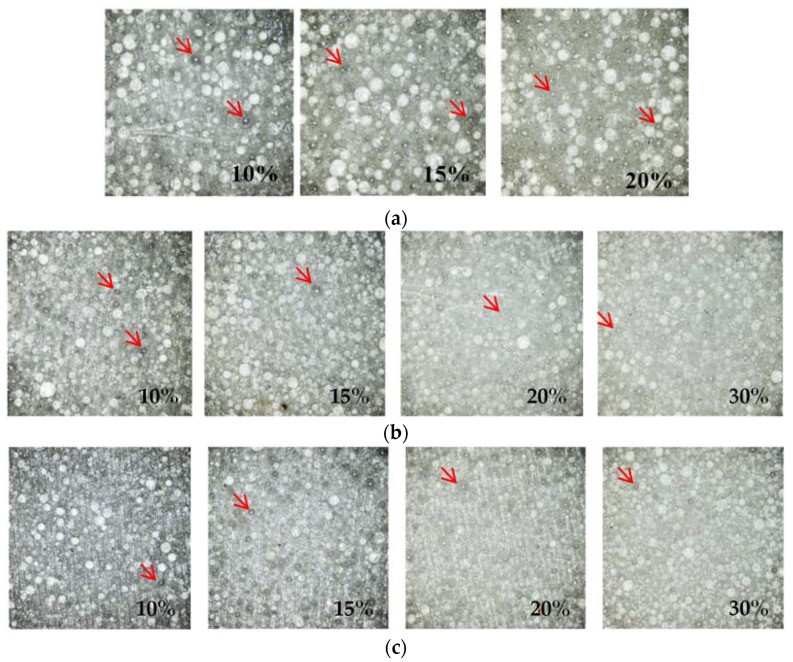
Microstructure of the epoxy/hollow glass microsphere composites. (**a**) BR20, (**b**) BR40, and (**c**) BR60 microsphere. (The value on the figure is the weight ratio *R*_c_, the red arrows are the air bubble).

The first matching layer was cut into the design shape to be pasted on the 1-3 piezoelectric composite. The surface after cutting was observed by the microscopy to check the microstructure. The white microsphere and epoxy matrix can be identified in all figures. Some white dots, marked by the arrows, are air bubbles. With the increase in the microsphere weight ratio (*R*_c_), the number of bubbles increased, based on the observation in Figure 5a. Figure 5b,c show a similar trend. The higher *R*_c_ increases the viscosity of the mixture, which makes the bubbles hard to escape. The material flowing in the high viscosity environment is also constrained, which makes the matching layer easy to inhomogeneous. The inhomogeneous microstructure of the matching layer will affect the longitudinal wave velocity, which can be another possible reason for the experiments BR20-20-15. The bubble and inhomogeneous material also enlarge the error between the theoretical model and experiments results, indicated in Table 3, which makes the acoustic properties of the matching layer difficult to predict. The diluent can improve the bubble release, as seen in the 15% and 20% *R*_c_ in Figure 5a and Table 3.

### 5.2. Validation of the Matching Layer Modeling

Table 3 also provides the modeling results of density, longitudinal wave velocity, and acoustic impedance. The proposed density model can predict all experiment density results with an error of less than 3%, which proves the correctness and accuracy of the modeling. For the longitudinal wave velocity, all experiments are modeled in the error of less than 6.5%, except BR20-20-15, which has an error of up to 10.9%. The acoustic impedance prediction results share a similar trend with the longitudinal wave velocity, which has high accuracy with an error of less than 6.3% if the BR20-20-15 is not considered. As mentioned above, in BR20, the viscosity of the composite increases tremendously when the weight ratio is larger than 15%, and the diluent has to be used. When 15% diluent is added in BR-20-15-15, the matching layer can be formed. However, when the weight ratio of hollow glass microspheres increases to 30%, the microsphere cannot be stirred to mix with the resin even with 15% diluent. More diluent, up to 50%, had been tried, which still can obtain many visible bubbles when the composite is cured. The reason for the relatively poor accuracy in BR20-20-15 maybe result from the inhomogeneous of the material and microbubble inside, which has been confirmed by Figure 5.

After verification of the matching layer modeling in this study, the best material components needed to form the theoretical acoustic impedance are predicted. Based on the proposed modeling, the theoretical first matching layer can be made by BR20 with a 19% weight ratio and 15% diluent. The fabricated sample from the modeling has a density of 625.35 kg/m^3^ and 2186 m/s acoustic velocity. The calculated acoustic impedance is 1.36 MRayl, which is a 1.4% error from the theoretical value. The second matching layer, which can be considered as the composition of polypropylene and air bubble, needs an expansion rate of 80 based on the modeling in this study. The density, acoustic velocity, and acoustic impedance of the fabricated second layer are 67.13 kg/m^3^, 738 m/s, and 0.049 MRayl, respectively. The larger acoustic impedance error results from the inaccuracy of the expansion rate, which is hard to control.

**Figure 6:**
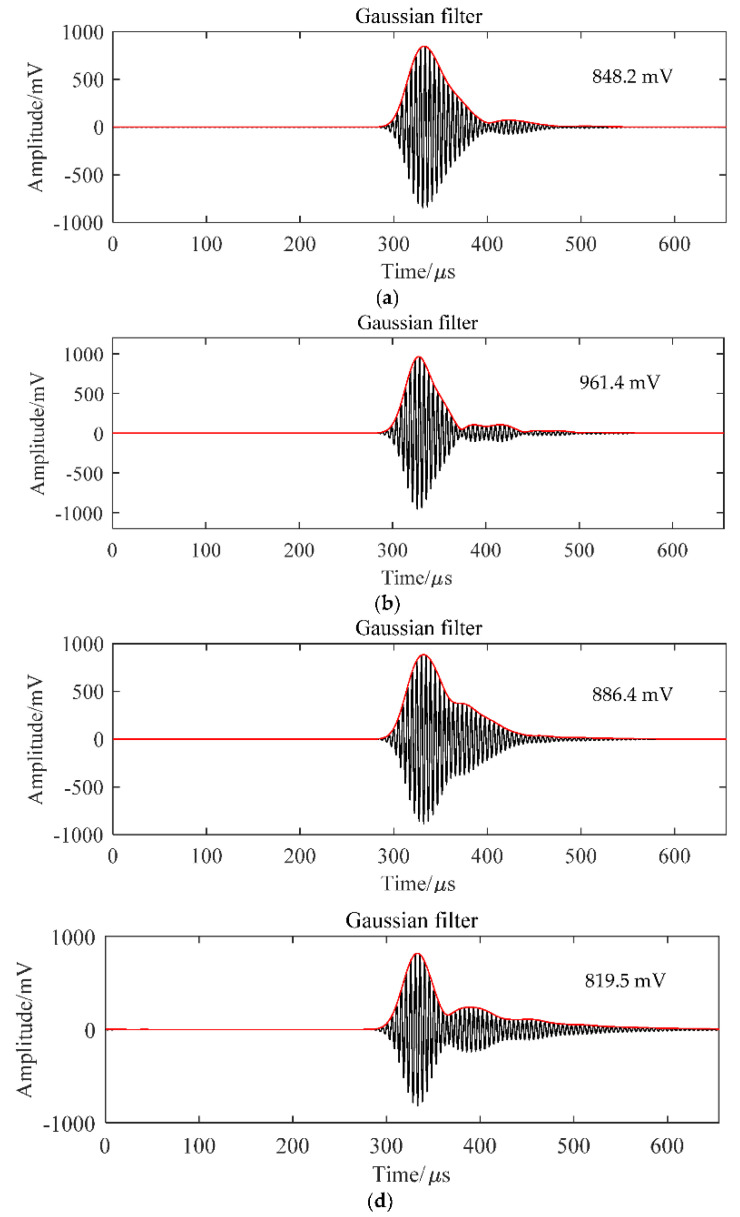
Time–domain filtered and enveloped signal of (**a**) BR20-15-15, (**b**) BR20-19-15, (**c**) BR20-15-15, and (**d**) transducer from Japan Probe Co., LTD.

To further verify the modeling results on the sensitivity, several matching layers were used to make the transducer to test the signal amplitude in the air by using the testing platform in Figure 4. Due to the small acoustic impedance in the second matching layer, the accurate controlling of acoustic impedance is difficult. Thus, only the first matching layer acoustic impedance changes in the experiments. The second matching layer used the 0.049 MRayl samples. All matching materials were sliced into 20 mm × 14 mm blocks with a quarter wavelength of the acoustic in matching material thickness and pasted on the 1-3 composite with the E51 epoxy to fabricate the transducer. From the modeling result, the first layer in BR20-19-15 has the acoustic impedance closest to the theoretical value. The other two matching layers with BR20-15-15 and BR20-20-15, with higher and lower acoustic impedance, respectively, were also tested. Moreover, another purchased transducer from Japan Probe was also used to compare the performance of the self-developed with the existing industry transducer. The received signal results are shown in Figure 6. The BR20-19-15 (seen in Figure 6b) has the highest amplitude, up to 961.4 mV, which is about 10% higher than BR 20-15-15 (Figure 6a) and BR 20-20-15 (Figure 6c), which are 848.2 and 886.4 mV, respectively. This phenomenon confirms that the higher or lower acoustic impedance compared to the theoretical acoustic impedance will result in a weaker amplitude and reduce the performance of the air-coupled transducer. Compared to the Japan Probe transducer with the same 200 kHz resonant frequency, the self-developed transducer has a 20% higher amplitude, which further proves the significance of this study.

### 5.3. Effect of Bonding Layer on Transducer Performance

**Figure 7:**
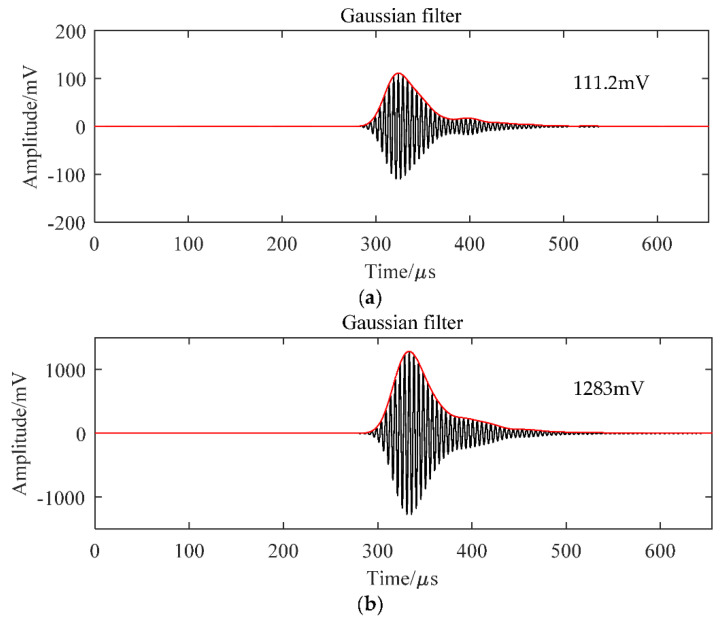
Time–domain filtered and enveloped signal. (**a**) Without bonding layer, (**b**) with the hollow glass beads/epoxy resin system.

The acoustic impedances of each layer with different bonding systems are shown in Table 4, and the amplitude signals are provided in Figure 7. Without the bonding layer, the 1-3 piezoelectric composite, first matching layer, and second matching layer are connected by the air, and the transducer has an amplitude of 111.2 mV, as demonstrated in Figure 7a. This amplitude is only 11.5% of the transducer bonded by the epoxy, as provided in Figure 6b. After changing the bonding material to the hollow glass microsphere/epoxy composite, the amplitude increased to 1283 mV, 33% higher than that of the epoxy bonding. This result can be explained by the acoustic impedance distribution in Table 4.

When the acoustic wave propagates from one homogenous material to another, both the reflection and refraction happen in the interface. The reflected acoustic wave returned to the first material, and the refracted one prorogates into the second material. The larger discrepancy between the two materials, the smaller percentage of the acoustic can propagate into the second material. For the transducer with two matching layers, there are five interfaces to be penetrated. When connected by air, most acoustic waves from the 1-3 piezoelectric reflect the composite, and only a few propagate into the air. When the acoustic wave in the air propagates to the first matching layer, most of the energy reflects into the air again, which happens several times in the air bonded transducer. Thus, little energy can pass through the transducer and be received by the receiving transducer. For the transducer bonded by the epoxy, the discrepancy is reduced, especially between the 1-3 composite and the first matching layer. This reduced acoustic impedance difference allows more energy to pass through the transducer. For the transducer bonded by the hollow glass microsphere and epoxy composite, even if the difference between the 1-3 piezoelectric composite and the first matching layer increased, the interface layer decreased to three to reduce the energy dissipation.

### 5.4. Practical Application Test of Self-Developed Air-Coupled Ultrasonic Transducer

**Figure 8:**
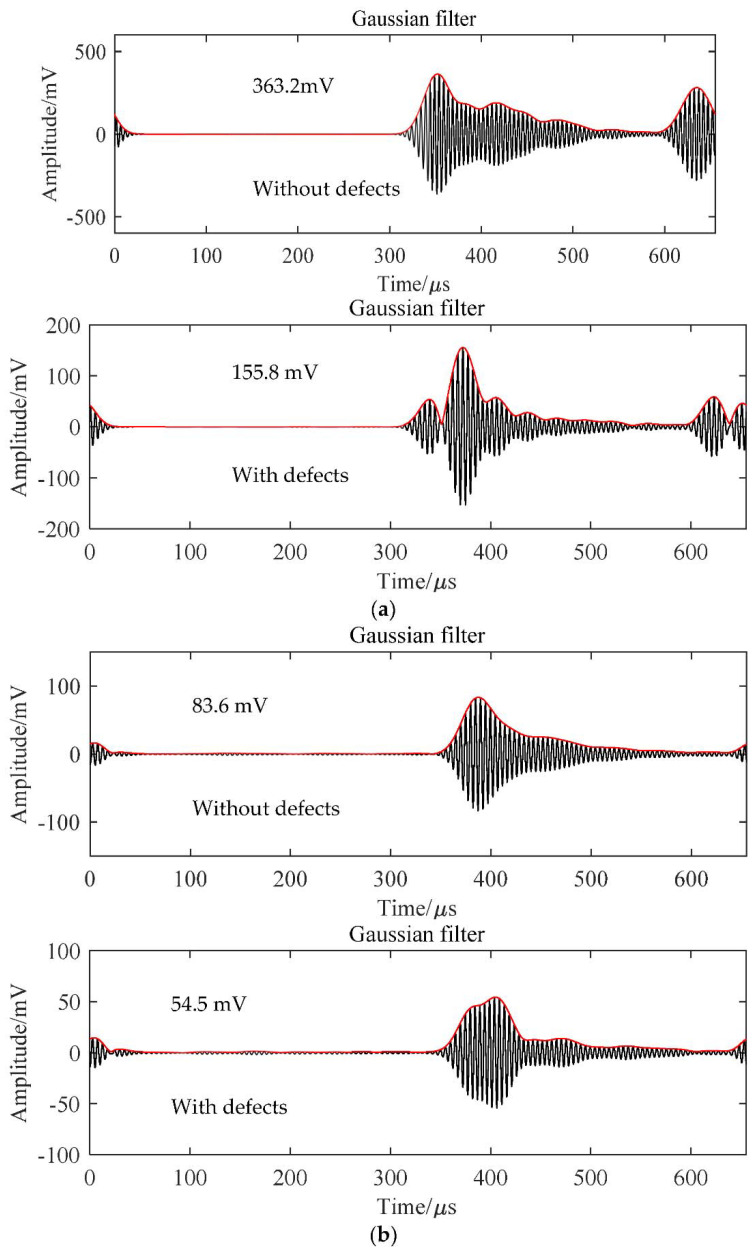
Time–domain filtered and enveloped signal of (**a**) PVC foam, (**b**) CFRP plates with or without defects.

The received signals from the air-coupled ultrasonic transducer after penetrating the testing plate are shown in Figure 8. When an ultrasonic wave propagates in PVC foam and CFRP plate without defects, the peak-to-peak amplitudes of received signals are respectively 363.2 and 83.6 mV, as seen in Figure 8a,b. When the defects in PVC foam and CFRP plates locate in the ultrasonic propagation path, the peak-to-peak amplitude of received signals were 155.8 and 54.5 mV, which decreased by about 57% and 35%, respectively. Due to the interaction between defects and ultrasonic waves, the energy of the transmitted wave was reduced, which led to the change in the shape and amplitude of the received wave. Due to the different types, sizes, and locations of defects, the received acoustic wave may change into various shapes, but this will not affect the detection of defects. By comparing the waveform amplitude of the received signals with or without defects, the defects are easy to detect. Further, the defect information can be directly distinguished by scanning and detecting the area to be inspected. The air-coupled ultrasonic transducer developed in this study can be used in practical testing applications.

## 6. Conclusions

This paper introduces a methodology to model, design, fabricate, and optimize air-coupled ultrasonic transducers matching system, which includes the matching layer and bonding layer. The matching layer modeling process and evaluation methods are demonstrated and verified by the self-developed air-coupled ultrasonic transducer with double matching layers. The mechanism of the matching layer components affecting the transducer performance is explained. Additionally, the bonding layer effects on the transducer performance are described. The testing results indicate that the self-developed air-coupled ultrasonic transducer in this study has a 20% higher amplitude than the product on the market and can easily identify defects in non-metallic materials. This study provides the foundation for air-coupled ultrasonic transducer modeling and development.

## Figures and Tables

**Table 1:** List of experiment parameters.

Material	Property	Value
1-3 type piezoelectric composite	Density, *ρ*_o_ (kg/m^3^)	5501.81
	Acoustic velocity, *c_L_* (m/s)	3521
	Acoustic impedance, *Z*_o_ (MRayl)	19.37
First matching layer	Hollow glass microspheres	BR20, BR40, and BR60
	Ratio of glass microspheres in weight, *k*	10%, 15%, 20%, and 30%
	Density of E51 resin, (kg/m^3^)	1168.33
	Density of curing agent, (kg/m^3^)	1036
	Density of diluent, (kg/m^3^)	907
	Acoustic velocity of pure E51 + curing agent, (m/s)	3124
	Acoustic velocity of diluent in liquid, (m/s)	1040
	Shrinkage ratio of pure E51 + curing agent	2%
Second matching layer	Density, *ρ*_2_ (kg/m^3^)	67.13
	Acoustic velocity, *c_L_*_2_ (m/s)	738
	Acoustic impedance, *Z*_2_ (MRayl)	0.049
Bonding layer	Material	Non, epoxy, epoxy/glass microsphere composite

**Table 2:** Hollow glass bead parameter index.

Hollow Glass Microsphere	Average Diameter, *d* (μm)	Real Density, *ρ*_R_ (kg/m^3^)	Bulk Density, *ρ*_B_ (kg/cm^3^)	Wall Thickness (μm)	Acoustic Velocity (m/s)
BR20	100	200	120	0.5–1	2280
BR40	85	400	240	1–2	
BR60	70	600	390	1.5–3.5	

**Table 3:** First matching layer acoustic properties. (Exp-experiment, Mod-modeling).

Model-*R*c(%)-*R*d(%)	Density, *ρ*_1_ (kg/m^3^)			Longitudinal Wave Velocity, *c_L_*_1_ (m/s)			Acoustic Impedance, *Z*_1_ (MRayl)		
	Exp	Mod	Error	Exp	Mod	Error	Exp	Mod	Error
BR20-10-0	813	788	3.0%	2770	2726	1.6%	2.25	2.15	4.5%
BR20-15-15	695	695	0.0%	2367	2357	0.4%	1.64	1.64	0.1%
BR20-20-15	609	614	0.8%	2114	2344	10.9% *	1.29	1.44	11.6% *
BR40-10-0	954	982	2.9%	2890	2864	0.9%	2.76	2.81	1.9%
BR40-15-0	929	908	2.2%	2841	2774	2.4%	2.64	2.52	4.6%
BR40-20-0	840	845	0.6%	2820	2701	4.2%	2.37	2.28	3.7%
BR40-30-0	742	742	0.1%	2770	2590	6.5%	2.05	1.92	6.3%
BR60-10-0	1059	1069	0.9%	2962	2931	1.1%	3.14	3.13	0.2%
BR60-15-0	1015	1025	1.0%	2857	2853	0.1%	2.9	2.92	0.8%
BR60-20-0	977	984	0.7%	2807	2786	0.8%	2.74	2.74	0.0%
BR60-30-0	896	911	1.6%	2686	2673	0.5%	2.41	2.43	1.0%

* The high error is due to the bubble and inhomogeneous microstructure in the matching layer.

**Table 4:** Acoustic impedance distribution of the transducer with three different bonding materials.

Structure Layer	Acoustic Impedance (MRayl)		
	Air	Epoxy	Hollow Glass Microsphere and Epoxy Composite
1-3 piezoelectric composite	19.37	19.37	19.37
First bonding layer	0.000425	3	1.36
First matching layer	1.36	1.36	1.36
Second bonding layer	0.000425	3	1.36
Second matching layer	0.049	0.049	0.049
Air	0.000425	0.000425	0.000425

## Data Availability

Data sharing not applicable.
